# Multi-centre reproducibility of diffusion MRI parameters for clinical sequences in the brain

**DOI:** 10.1002/nbm.3269

**Published:** 2015-03-19

**Authors:** Matthew Grech-Sollars, Patrick W Hales, Keiko Miyazaki, Felix Raschke, Daniel Rodriguez, Martin Wilson, Simrandip K Gill, Tina Banks, Dawn E Saunders, Jonathan D Clayden, Matt N Gwilliam, Thomas R Barrick, Paul S Morgan, Nigel P Davies, James Rossiter, Dorothee P Auer, Richard Grundy, Martin O Leach, Franklyn A Howe, Andrew C Peet, Chris A Clark

**Affiliations:** a Developmental Imaging and Biophysics Section, UCL Institute of Child Health, University College LondonLondon, UK; b CR UK and EPSRC Cancer Imaging Centre, Institute of Cancer Research and Royal Marsden Foundation TrustBelmont, Surrey, UK; c Division of Clinical Sciences, St George's, University of LondonLondon, UK; d Division of Clinical Neuroscience, School of Medicine, University of NottinghamNottingham, UK; e The Children‘s Brain Tumour Research Centre, University of NottinghamNottingham, UK; f School of Cancer Sciences, University of BirminghamBirmingham, UK; g Department of Radiology, Great Ormond Street Hospital for ChildrenLondon, UK; h Imaging and Medical Physics, University Hospitals Birmingham NHS Foundation TrustBirmingham, UK; i Electrical and Computer Engineering, University of BirminghamBirmingham, UK

**Keywords:** diffusion, MRI, reproducibility, brain, multi-centre

## Abstract

The purpose of this work was to assess the reproducibility of diffusion imaging, and in particular the apparent diffusion coefficient (ADC), intra-voxel incoherent motion (IVIM) parameters and diffusion tensor imaging (DTI) parameters, across multiple centres using clinically available protocols with limited harmonization between sequences.

An ice–water phantom and nine healthy volunteers were scanned across fives centres on eight scanners (four Siemens 1.5T, four Philips 3T). The mean ADC, IVIM parameters (diffusion coefficient *D* and perfusion fraction *f*) and DTI parameters (mean diffusivity MD and fractional anisotropy FA), were measured in grey matter, white matter and specific brain sub-regions. A mixed effect model was used to measure the intra- and inter-scanner coefficient of variation (CV) for each of the five parameters.

ADC, *D*, MD and FA had a good intra- and inter-scanner reproducibility in both grey and white matter, with a CV ranging between 1% and 7.4%; mean 2.6%. Other brain regions also showed high levels of reproducibility except for small structures such as the choroid plexus. The IVIM parameter *f* had a higher intra-scanner CV of 8.4% and inter-scanner CV of 24.8%. No major difference in the inter-scanner CV for ADC, *D*, MD and FA was observed when analysing the 1.5T and 3T scanners separately.

ADC, *D*, MD and FA all showed good intra-scanner reproducibility, with the inter-scanner reproducibility being comparable or faring slightly worse, suggesting that using data from multiple scanners does not have an adverse effect compared with using data from the same scanner. The IVIM parameter *f* had a poorer inter-scanner CV when scanners of different field strengths were combined, and the parameter was also affected by the scan acquisition resolution. This study shows that the majority of diffusion MRI derived parameters are robust across 1.5T and 3T scanners and suitable for use in multi-centre clinical studies and trials. © 2015 The Authors *NMR in Biomedicine* Published by John Wiley & Sons Ltd.

## Introduction

Diffusion imaging is widely used both in research and in the clinic. In areas where clinical data sets are sparse, such as paediatric oncology or other rare diseases, it may be necessary to collate data from multiple centres in order to conduct a sufficiently powered analysis. While the availability of multi-centre data may be beneficial in terms of increasing the amount of data available for a given study, it introduces the question of whether data obtained using clinical sequences on different scanners with different field strengths is comparable. This question also arises in longitudinal studies, where patients may be scanned on the same scanner as used previously or a different one. We thus aim to validate clinical diffusion imaging measurements across multiple centres and on different scanners.

The basis of this study was to determine the reproducibility of diffusion measures, commonly used in multisite clinical research studies, on both a phantom and a group of volunteers, each being scanned at multiple sites and on different scanners. The centres that took part are part of a multi-centre study of cancer imaging in children, and protocols for imaging had previously been agreed and made available. However, the need to integrate imaging research with clinical MRI examinations being carried out in the patients and the need to utilize historically acquired data necessitated a significant degree of flexibility in implementing the protocols locally, so that for example there were significant differences, in particular, in the diffusion-weighted imaging (DWI) protocols used between centres. In this study DWI (1) and diffusion tensor imaging (DTI) (2) parameters were analysed. More specifically the reproducibility of the mono-exponential fit to DWI (the apparent diffusion coefficient, ADC), the bi-exponential fit to DWI as applied through intra-voxel incoherent motion (IVIM) (3), and the mean diffusivity (MD) and fractional anisotropy (FA) obtained from DTI datasets were investigated.

While previous studies have been carried out to examine the reproducibility of diffusion imaging parameters at a single centre (4–6) and at multiple centres (7–12), most studies aimed to match the imaging sequence used across all scanners. Two of these studies were carried out on a mixture of 1.5T and 3T scanners, with one study analysing the reproducibility of ADC (8) and one study analysing the reproducibility of FA (10). The aim of this study was to quantify the reproducibility of DWI and DTI parameters acquired with sequences in routine clinical use without extensive harmonization across scanners.

## Materials and Methods

### Volunteers

Nine healthy volunteers (seven male, two female; aged 25–34 years at first scan; mean 29 years) were enrolled in this multi-centre study. Ethical approval was given by the research ethics committee and informed consent was obtained at all centres. All data was anonymized in accordance with the Data Protection Act, UK.

### Scanners

Eight scanners (three Siemens Avanto 1.5T, one Siemens Symphony 1.5T and four Philips Achieva 3T), across five centres, were used in the study. Between four and eight volunteers were scanned on each scanner. One volunteer on scanners C, D and E and two volunteers on scanners A, B, F, G and H had repeat scans performed on a different date from their first scan. All scans were acquired over a period of 18 months and a total of 65 imaging sessions took place across all centres.

### Phantom

An ice–water phantom (13) was scanned on all scanners. Different fluids will have a varying ADC value, which also varies with temperature. ADC in water has been measured as between 1.756 × 10^−3^ mm^2^ s^−1^ at 15 °C and 2.616 × 10^−3^ mm^2^ s^−1^ at 30 °C (14). The advantage of using ice–water is that the temperature is fixed, and is not affected by the temperature inside the scanner room. Ice–water is expected to have an ADC of 1.099 × 10^−3^ mm^2^ s^−1^ (15). The phantom consisted of five tubes filled with distilled water and one tube filled with sucrose. The phantom was filled with ice and cold water, and was allowed to settle for an hour so as to reduce the temperature of the fluid-filled tubes to 0 °C and reach thermal equilibrium. Measurements were taken from the water-filled tubes.

### Imaging protocol

The clinical protocols from the five centres were acquired on eight scanners for DWI (Table1) and seven scanners for DTI (Table2). Additional *b*-values of 0, 50, 100, 300, 500, 600 and 1000 were acquired for the DWI protocol at all centres. In addition, a high resolution *T*_1_-weighted image was acquired at each centre.

**Table 1:** Clinical DWI protocol

	Great Ormond Street Hospital for Children NHS Trust		St George's Hospital	The Royal Marsden NHS Foundation Trust		Nottingham University Hospital NHS Trust	Birmingham Children's Hospital	
Scanner	A	B	C	D	E	F	G	H
Manufacturer	Siemens	Siemens	Philips	Siemens	Philips	Philips	Siemens	Philips
Model	Avanto	Symphony	Achieva	Avanto	Achieva	Achieva	Avanto	Achieva
Field strength (T)	1.5	1.5	3	1.5	3	3	1.5	3
Head coil channels	12	8	32	12	8	8	8	32
*T*_R_ (ms)	2700	3500	3800	3800	3800	3280	4400	3800
*T*_E_ (ms)	96	109	74	73	73	73	89	74
*b*-values	0, 500, 1000	0, 500,1000	0, 1000	0, 50, 100, 300, 600, 1000	0, 50, 100, 300, 600, 1000	0, 1000	0, 1000	0, 1000
FOV (mm)	230 × 230	230 × 230	230 × 230	230 × 230	230 × 230	224 × 224	230 × 230	230 × 230
No of slices	19	19	22	22	22	32	28	22
Slice thickness (mm)	5	5	5	5	5	4	5	5
Acquired matrix	128 × 128	128 × 128	128 × 128	128 × 128	128 × 128	112 × 112	192 × 192	128 × 128
Interpolated matrix	128 × 128	256 × 256	256 × 256	256 × 256	256 × 256	224 × 224	192 × 192	256 × 256
Orientation	axial	axial	axial	axial	axial	axial	axial	axial
Bandwidth (Hz px^−1^)	1502	1500	2308	1860	1860	2441	1240	2307
Parallel imaging	2	none	2	2	2	2	2	2
NSA	3	2	3	3	3	2	1	3
Resolution (mm)	1.8 × 1.8 × 5	1.8 × 1.8 × 5	1.8 × 1.8 × 5	1.8 × 1.8 × 5	1.8 × 1.8 × 5	2 × 2 × 4	1.2 × 1.2 × 5	1.8 × 1.8 × 5
Voxel size (mm)	1.8 × 1.8 × 5	0.9 × 0.9 × 5	0.9 × 0.9 × 5	0.9 × 0.9 × 5	0.9 × 0.9 × 5	1 × 1 × 4	1.2 × 1.2 × 5	0.9 × 0.9 × 5

**Table 2:** Clinical DTI protocol

	Great Ormond Street Hospital for Children NHS Trust	St George's Hospital	The Royal Marsden NHS Foundation Trust		Nottingham University Hospital NHS Trust	Birmingham Children's Hospital	
Scanner	A	C	D	E	F	G	H
Manufacturer	Siemens	Philips	Siemens	Philips	Philips	Siemens	Philips
Model	Avanto	Achieva	Avanto	Achieva	Achieva	Avanto	Achieva
Field strength(T)	1.5	3	1.5	3	3	1.5	3
Head coil channels	12	32	12	8	8	8	32
*T*_R_ (ms)	7300	6000	7300	7767	6268	6510	6000
*T*_E_ (ms)	81	70	81	70	53	86	70
*b*-values	0, 1000	0, 1000	0, 1000	0, 1000	0, 1000	0, 1000	0, 1000
FOV (mm)	240 × 240	240 × 240	240 × 240	240 × 240	240 × 240	240 × 231	240 × 240
No of slices	60	48	60	70	56	51	48
Slice thickness (mm)	2.5	2.5	2.5	2	2.5	2.5	2.5
Matrix	96 × 96	96 × 96	96 × 96	128 × 128	96 × 96	108 × 104	96 × 96
Bandwidth (Hz px^−1^)	1447	3324.3	1447	1783	3321	1493	3324
Parallel imaging	2	2	2	2	2	2	2
NSA	1	1	1	2	1	2	1
Gradient directions	60	32	30	32	15	20	32
*b*-zeros	3	1	5	1	1	1	1
Voxel size (mm)	2.5 × 2.5 × 2.5	2.5 × 2.5 × 2.5	2.5 × 2.5 × 2.5	1.875 × 1.875 × 2	2.5 × 2.5 × 2.5	2.2 × 2.2 × 2.5	2.5x2.5x2.5

### Data analysis

#### Segmentation

In volunteers, DWI and DTI parameters were measured in total grey and white matter. For DWI parameters, the masks for total grey and white matter were created by segmenting the *b*_0_ image using SPM (16) and a probability threshold of 0.95. For the DTI parameters, the masks were created by segmenting the *S*_0_ image – the estimated *b*_0_ image output by FSL (17).

**Figure 1:**
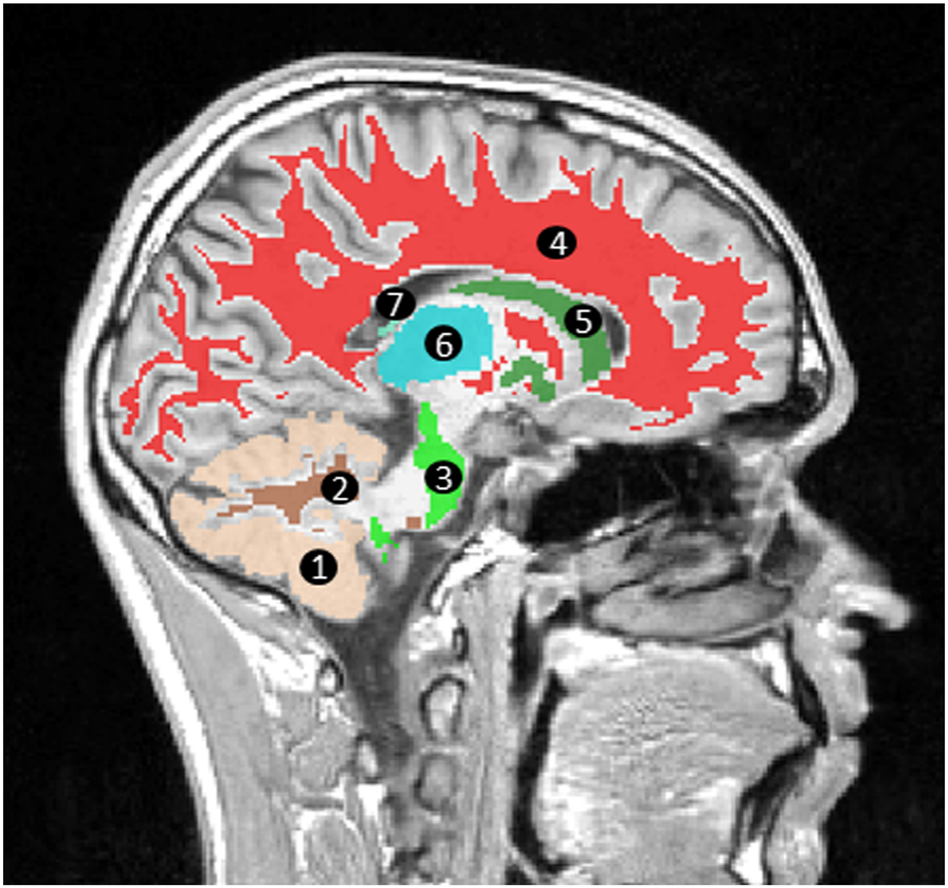
Segmentation of *T*_1_-weighted image using FreeSurfer. *T*_1_-weighted images were segmented using FreeSurfer in order to create masks defining 1, cerebellar cortex, 2, cerebellar white matter, 3, brain stem, 4, cerebral white matter, 5, basal ganglia (including the caudate nucleus, the putamen and the globus pallidus), 6, thalamus, 7, choroid plexus, and the optic chiasm (not shown).

DWI and DTI parameters were also measured in eight brain regions selected for their vulnerability to neurological diseases and conditions seen in the clinic, with a particular emphasis on regions affected by paediatric brain tumours. The regions studied included cerebellar white matter, cerebellar grey matter, brain stem, cerebral white matter, basal ganglia, thalamus, choroid plexus and the optic chiasm (shown in Fig.1). The masks for the selected brain regions were created by segmenting high-resolution *T*_1_-weighted images using FreeSurfer (18–21). High-resolution *T*_1_-weighted images were not available for all volunteers on scanner D and on one volunteer on scanner G and hence these were excluded from the analysis. Furthermore, the segmentation failed on one volunteer on scanner H. Thus the analysis was conducted on 56 imaging sessions for DWI and on 47 imaging sessions for DTI. The mean values for the DWI parameters in these regions were calculated by registering the *b*_0_ image to the high-resolution *T*_1_-weighted image, then subsequently applying the same transformation to ADC, *D* and *f*. Similarly, the mean values for the DTI parameters in these regions were calculated by registering the *S*_0_ image to the high-resolution *T*_1_-weighted image and subsequently applying the same transformation to MD and FA. An image erosion process of one voxel was used on the masks output by FreeSurfer prior to applying these to the registered DWI and DTI parameters in order to avoid partial volumes. All registration was performed using an affine 12-parameter model with tri-linear interpolation in FLIRT, the linear image registration tool provided by FSL (22).

#### Apparent diffusion coefficient

The ADC was calculated from Equation [1]using custom written MATLAB scripts to determine the gradient to the graph of 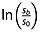 against b, by applying a linear fit through the clinical *b*-values shown in Table1.


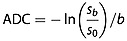
[1]

where *s*_*b*_is the signal at the specific *b*-value, and *s*_0_ is the signal at *b*_0_.

In the phantom, the ADC was calculated in the ice–water tubes by manually drawing an aggregate region of interest (ROI) over the tubes, avoiding boundary regions to include only areas of water, and calculating the mean ADC over the area and across all water-filled tubes. In volunteers, the segmented images were used as masks to calculate the mean ADC in grey matter, white matter and the eight selected brain regions.

#### Intra-voxel incoherent motion

DWI data were processed using all *b*-values measured (*b* = 0, 50, 100, 300, 500, 600 and 1000) and the IVIM model (3), a method that assumes that two diffusing species give rise to the observed signal during *in vivo* DWI. These are the incoherent flow of blood–water in the randomly orientated micro-vascular network (referred to as fast, micro-circulation-driven pseudo-diffusion), and the molecular, thermally driven diffusion of water molecules in the extra-vascular space. Using this model, the observed signal intensity (*S*) at a given level of diffusion weighting (*b*) is given by


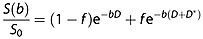
[2]

where *s*_0_ is the signal intensity without diffusion-weighting, *D* is the diffusion coefficient of water molecules in the tissue, D* is the fast pseudo-diffusion coefficient and *f* is the fraction of the total DWI signal that arises from the latter compartment.

The fitted parameters (*f*, *D* and D*) were obtained in a stepwise-sequential manner, due to limitations in the precision of fitting Equation [2]directly to DWI data (23). First, linear regression of ln(*S*/*S*_0_) versus *b* was used to obtain *D*, using only data acquired with *b* ≥ 300 s mm^−2^, at which the fast diffusing component (D*) is negligible due to the dephasing caused by the diffusion gradients. Raw data from all *b*-values were then used to fit *f* and D* (with *D* fixed), using an iterative Nelder–Mead nonlinear least squares algorithm. The mean values of *D* and *f* for both phantom and volunteers were derived through the same masks as used for measuring the mean ADC.

#### Diffusion tensor imaging

**Figure 2:**
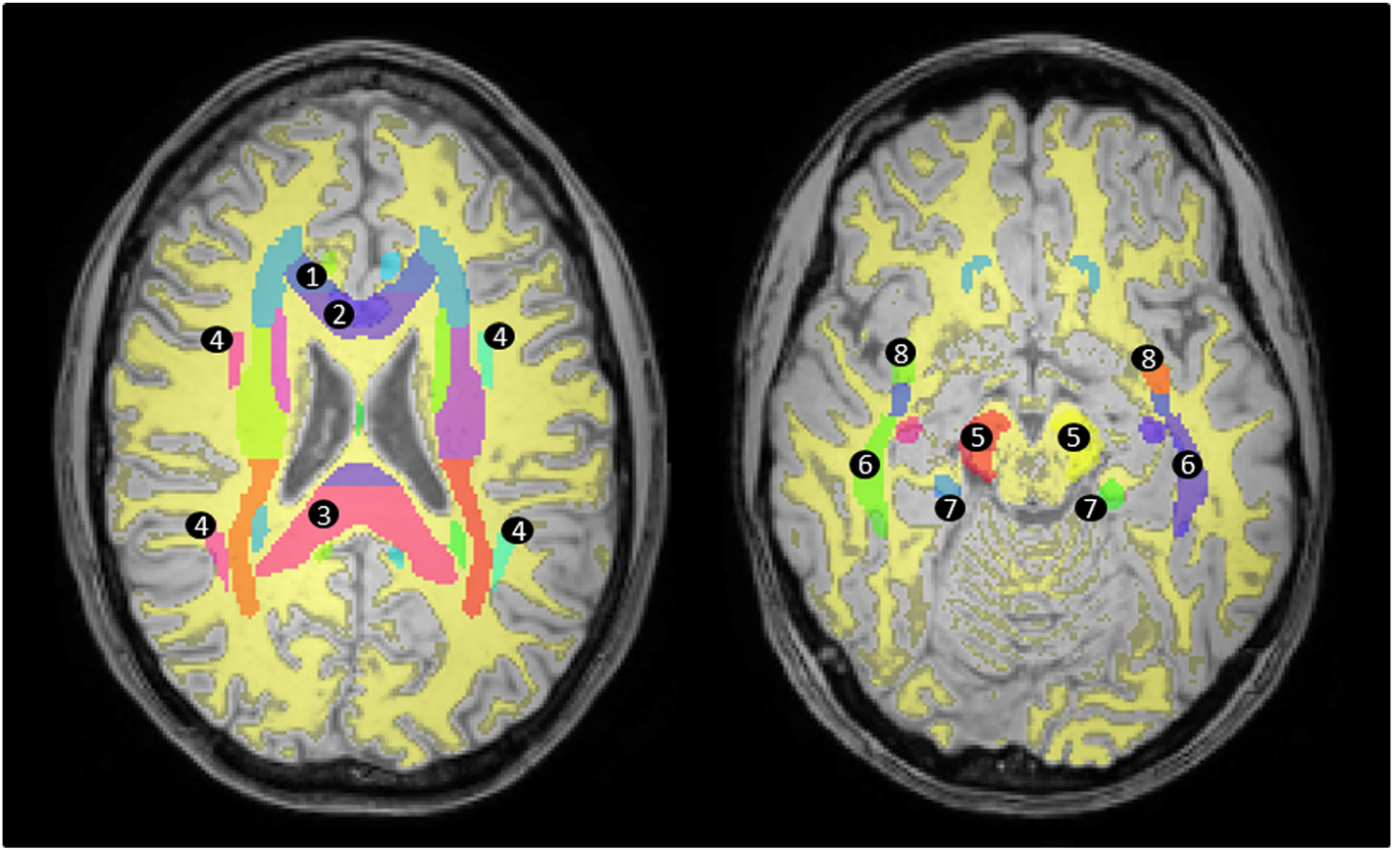
Measuring FA in white matter areas. The segmented white matter mask (yellow) is shown overlaid on a high-resolution *T*_1_-weighted image registered to standard MNI space. The ICBM-DTI-81 atlas (coloured areas) was used in order to measure the FA in areas defined as white matter according to the segmented mask in specific tracts: 1, genu of the corpus callosum; 2, body of the corpus callosum; 3, splenium of the corpus callosum; 4, superior longitudinal fasciculus; 5, cerebral peduncle; 6, sagittal stratum; 7, cingulum; 8, uncinate fasciculus.

MD and FA were calculated through dtoa software (24), which uses FSL (17) to compute the DTI parameters following eddy current correction. In the phantom the MD and FA in the ice–water tubes were calculated by manually drawing an ROI over the tubes, excluding boundary areas, and calculating the mean MD and FA in these areas. In the volunteers, similarly to ADC, segmented images were used to calculate the mean MD and FA in grey matter, white matter and the eight brain regions described. In addition, for the FA analysis, the ICBM-DTI-81 atlas (25) available in FSL was used to measure the mean FA in specific white matter ROIs shown in Figure2. This was done by first registering the *S*_0_ image to standard MNI space, then performing the same registration to the derived FA map, and then segmenting the *S*_0_ image to obtain a mask for the white matter. The mean FA in the ROIs was determined by overlaying the white matter mask and the atlas to the registered FA map, shown in Figure2.

### Statistical analysis

Statistical analysis on the phantom data consisted of measuring the coefficient of variation (CV), as defined by Equation [3], using the group mean and standard deviation of each measured mean ADC value in ice–water, across all scanners.


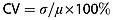
[3]

where *σ* is the standard deviation and *μ* is the mean.

Statistical analysis for the volunteer data was conducted using R software (26) and the lme4 package therein (27). In order to calculate the reproducibility of the above-mentioned parameters, a mixed effect model was used. The volunteer was considered as a random effect and the scanner considered as a fixed effect. Data from all time points, including repeat scans, were entered into the model. The mixed effect model gives a mean and standard deviation for the fixed effects, together with the standard deviation expected for the random effect and an error term that is considered to be the variation that can be expected in addition to both random and fixed effects. The CV was then calculated by fixing the value of the mean as measured from the model and then using the standard deviation for the fixed effects, random effects and error term in order to measure the reproducibility of the given measures across different scanners (the inter-scanner CV), across different volunteers (inter-volunteer) and irrespective of volunteer or scanner (the intra-scanner CV) respectively. The model was also constructed separately for 1.5T and 3T scanners in order to study whether there is any major difference in reproducibility between the two field strengths.

In addition, the inter-class correlation coefficient (ICC_inter_) and the intra-class correlation coefficient (ICC_intra_) were calculated from the inter- and the intra-scanner standard deviations as shown in Equations [4] and [5].


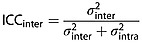
[4]


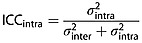
[5]

where *σ*_inter_ is the inter-scanner standard deviation and *σ*_intra_ is the intra-scanner standard deviation. These represent the proportion of the variance which is attributed to the inter- and the intra-scanner variations(12).

## Results

### Phantom

**Figure 3:**
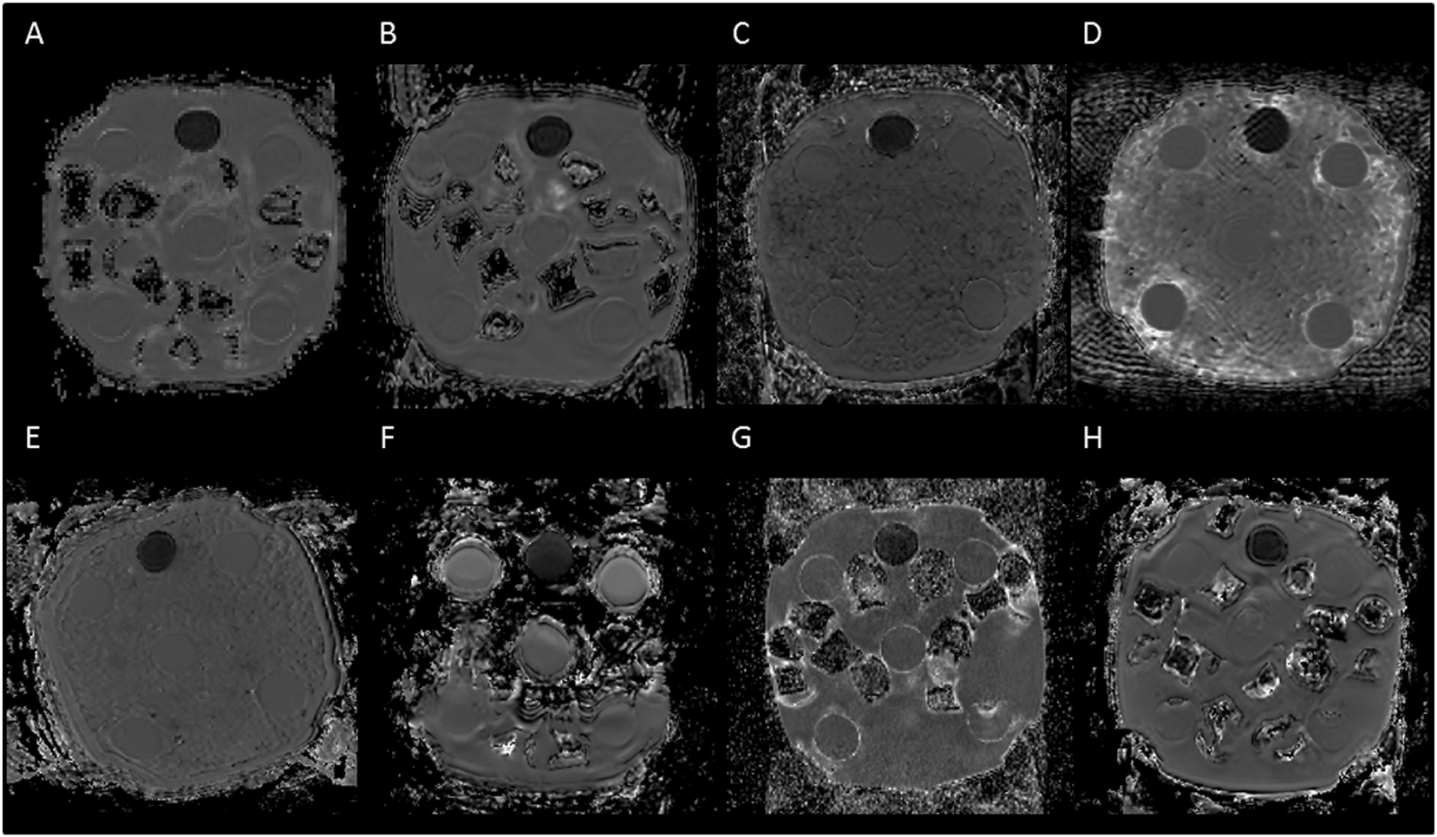
Comparison of the ice–water phantom ADC images across scanners. Images of the phantom from each of the scanners using the same contrast range are shown. The tube filled with sucrose appears darker, while the five tubes filled with distilled water are evenly separated from each other and can be seen to be surrounded by ice–water. The ice–water was prepared by using either ice cubes (e.g. scanner A) or crushed ice (e.g. scanner C). The protocol for scanning the phantom was not adhered to for scanner F, with the image showing that ice–water was not surrounding all of the tubes at acquisition.

Results for the ice–water phantom are shown in Table3 together with comparison images shown in Figure3. Values for ADC, *D* and MD were very similar. The team imaging with scanner F confirmed that when the phantom was scanned it was not given enough time to reach thermal equilibrium at 0 °C and consequently produced higher values than in the other scanners. Hence, calculations were also done excluding this scanner, which improved the CV for ADC, *D* and MD to 0.7, 1.4 and 0.9% respectively. FA and *f* had a high CV in the phantom. It was noted that *f*, at a value of 0.0591, was much higher for scanner G compared with all other scanners. Excluding scanner G and scanner F, the mean estimated value of *f* in the phantom was 0.0120, with a standard deviation of 0.0033 and a CV of 27.3%.

**Table 3:** Reproducibility of the ice–water phantom. The table shows the expected and mean ADC, *D*, *f*, MD and FA values for the ice–water phantom together with the associated standard deviation. The CV was computed and shown for each of these parameters

		DWI		IVIM				DTI			
		ADC mean ± std		D mean ± std		f mean ± std		MD mean ± std		FA mean ± std	
		×10^−3^ mm^2^ s^−1^		×10^−3^ mm^2^ s^−1^				×10^−3^ mm^2^ s^−1^			
Expected		1.099		1.099		≈0		1.099		≈0	
Scanner	A	1.1103	0.0214	1.1041	0.0275	0.0114	0.0097	1.1030	0.0177	0.0226	0.0177
	B	1.1116	0.0218	1.1148	0.0258	0.0115	0.0102	—	—	—	—
	C	1.1064	0.1070	1.1010	0.0274	0.0181	0.0137	1.0966	0.0271	0.0392	0.0149
	D	1.0971	0.0173	1.1060	0.0211	0.0081	0.0087	1.0921	0.0176	0.0240	0.0177
	E	1.1106	0.0191	1.1218	0.0169	0.0120	0.0141	1.1191	0.0315	0.0473	0.0234
	F	1.1525	0.0991	1.2059	0.0930	0.0229	0.0189	1.1884	0.0344	0.0488	0.0247
	G	1.1092	0.0914	1.1428	0.1147	0.0591	0.0451	1.0989	0.0240	0.0370	0.0145
	H	1.1223	0.0325	1.1346	0.0316	0.0110	0.0096	1.1116	0.0206	0.0405	0.0180
Overall		1.1150	0.0167	1.1289	0.0345	0.0193	0.0168	1.1157	0.0334	0.0371	0.0103
CV		1.5%		3.1%		87.1%		3.0%		27.8%	

### Volunteers

**Figure 4:**
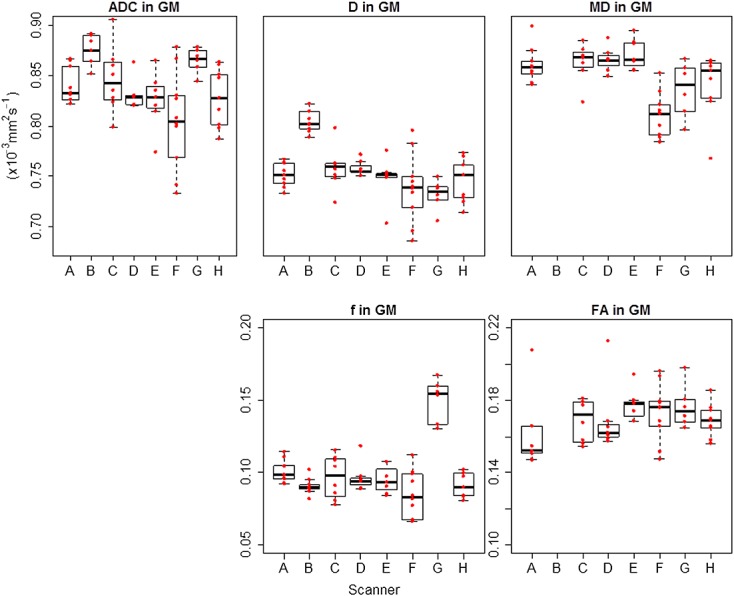
Box plots for DWI and DTI parameters across all scanners in grey matter (GM). ADC, *D* and MD are shown with the same range on the *y*-axis for direct comparison. A–H represent each scanner involved in the study, and the red data points represent individual subjects. ADC and MD had very similar values, while *D* had comparable but lower values. The boxplots confirm the higher values of *f* in scanner G.

**Figure 5:**
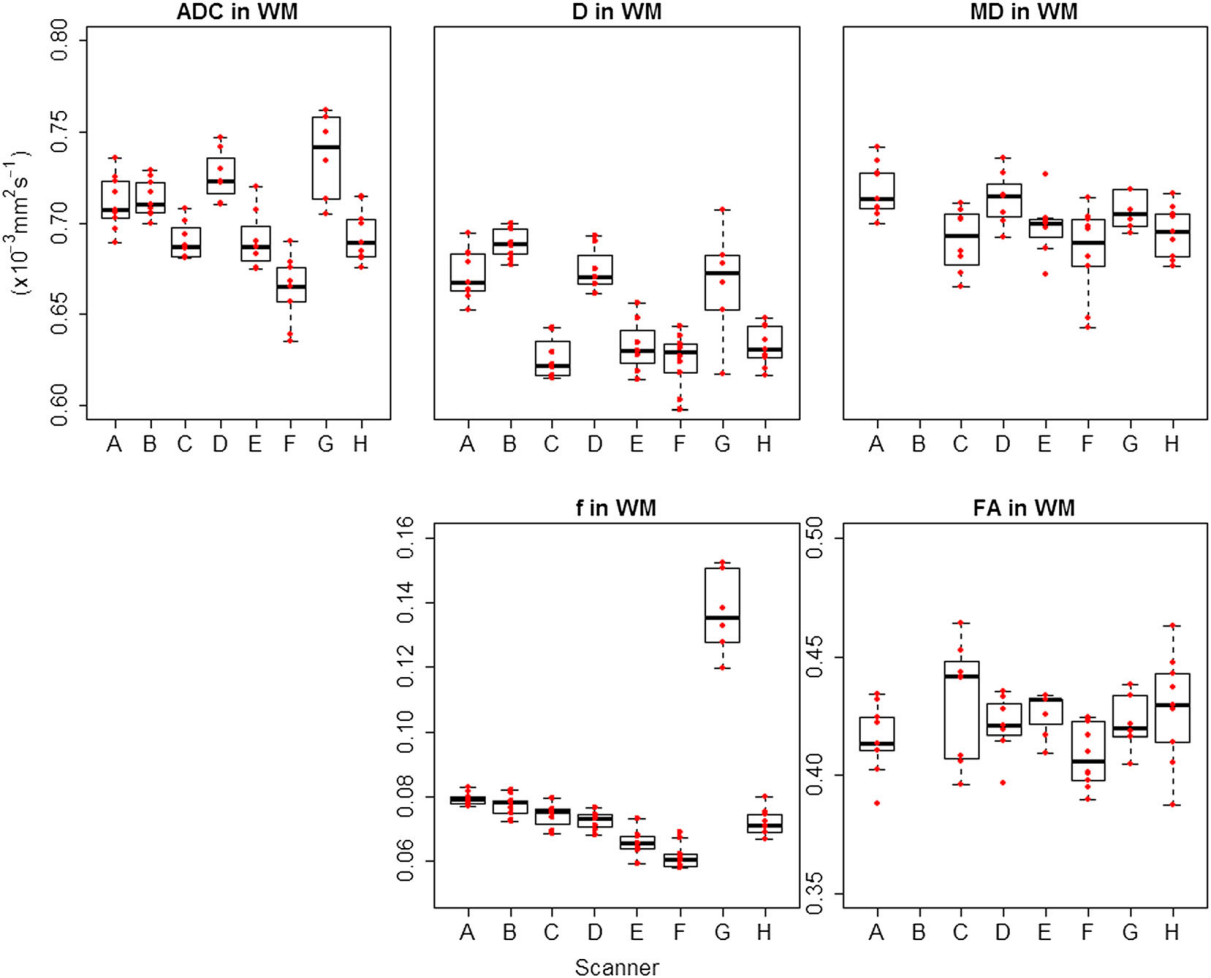
Box plots for DWI and DTI parameters across all scanners in white matter (WM). ADC, *D* and MD are shown with the same range on the *y*-axis for direct comparison. A–H represent each scanner involved in the study, and the red data points represent individual subjects. ADC and MD had very similar values, while *D* had comparable but lower values. The boxplots confirm the higher values of *f* in scanner G.

**Figure 6:**
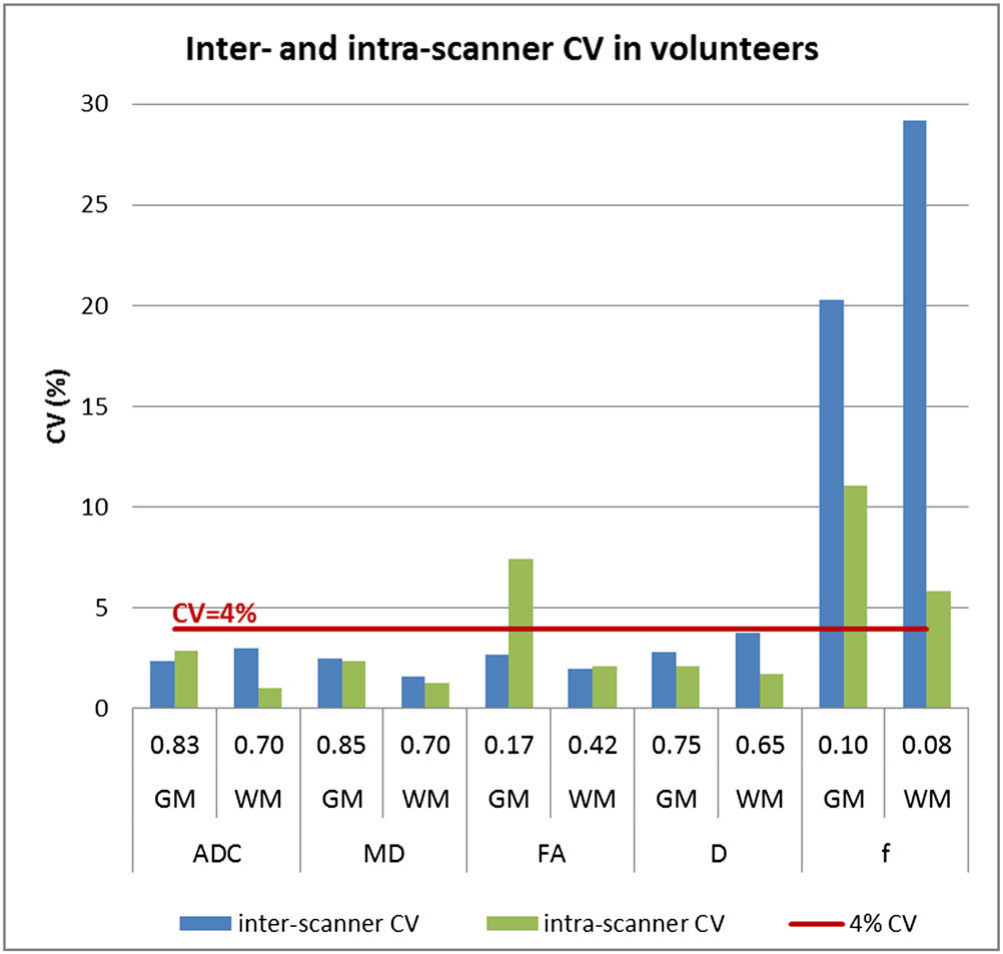
Inter- and intra-scanner CV in volunteers. The graph shows the inter- and intra-scanner CV for all parameters in overall grey matter (GM) and overall white matter (WM), together with the mean values for each of the parameters. The inter-scanner CV was less than 4% for all parameters except for the perfusion fraction. The higher intra-scanner CV for FA in grey matter is likely to be due to an increased noise effect on FA values closer to zero, driving the intra-scanner variation up.

Box plots showing the range of values observed in all volunteers across all scanners are shown for grey matter in Figure4 and for white matter in Figure5. Results for reproducibility of ADC, *D*, *f*, MD and FA in grey matter and white matter (depicted in Figure6) and the eight brain regions for volunteer scans are given in Tables4–8 respectively. The tables show the mean, standard deviation and CV results from the mixed-effect model describing the variation expected if the same volunteer is scanned on a different scanner (inter-scanner reproducibility), if a different volunteer is scanned on the same scanner (inter-volunteer) and if the same volunteer is scanned on the same scanner (intra-scanner reproducibility).

**Table 4:** Reproducibility in volunteers for ADC. The table shows the mean and standard deviation values for the measured ADC together with the associated CV in grey matter (GM) and white matter (WM) in all scans, and in eight separate regions in 56 of the scans. The first column gives the mean – over all scanners and volunteers as measured by the model. The second column shows the inter-scanner variability – the variation expected if the same volunteer (intra-volunteer) is scanned on a different scanner. The third column shows the inter-volunteer variability – the variation expected if a different volunteer is scanned on the same scanner (intra-scanner). The fourth column shows the intra-scanner variability – the variation expected if the same volunteer (intra-volunteer) is scanned on the same scanner. The last two columns show ICC_inter_ and ICC_intra_. An ICC_inter_ of 0.5 implies that the variations due to the inter-scanner effects are equal to the variations due to the intra-scanner effects. Values that are not estimated (NE) imply that the level of variation was not sufficiently large to warrant inclusion of the random effect in the analysis, as explained in the software manual in Reference (36)

Volunteers				Mean	Inter-scanner (intra-volunteer)	Inter-volunteer (intra-scanner)	Intra-scanner (intra-volunteer)	ICC_inter_	ICC_intra_
DWI – ADC	Overall	GM	×10^−3^ mm^2^ s^−1^	0.8327	±0.0203	±0.0246	±0.0242	0.41	0.59
			CV		2.4%	3.0%	2.9%		
		WM	×10^−3^ mm^2^ s^−1^	0.7010	±0.0210	±0.0156	±0.0072	0.90	0.10
			CV		3.0%	2.2%	1.0%		
	Cerebellum	Cortex (GM)	×10^−3^ mm^2^ s^−1^	0.8322	±0.0914	±0.0207	±0.0290	0.77	0.23
			CV		11.0%	2.5%	3.5%		
		WM	×10^−3^ mm^2^ s^−1^	0.7244	±0.099	±0.0137	±0.0429	0.84	0.16
			CV		13.7%	1.9%	5.9%		
	Brain stem	All	×10^−3^ mm^2^ s^−1^	0.8710	±0.1240	NE	±0.0679	0.91	0.09
			CV		14.2%	NE	7.8%		
	Cerebrum	WM	×10^−3^ mm^2^ s^−1^	0.7346	±0.0590	±0.0113	±0.0190	0.92	0.08
			CV		8.0%	1.5%	2.6%		
		Basal ganglia	×10^−3^ mm^2^ s^−1^	0.7574	±0.0590	±0.0162	±0.0293	0.87	0.13
			CV		10.6%	2.1%	3.9%		
		Thalamus	×10^−3^ mm^2^ s^−1^	0.7894	±0.0809	±0.0200	±0.0236	0.40	0.60
			CV		10.2%	2.5%	3.0%		
		Choroid plexus	×10^−3^ mm^2^ s^−1^	1.9049	±0.5502	±0.0920	±0.2103	0.91	0.09
			CV		28.9%	4.8%	11.0%		
		Optic chiasm	×10^−3^ mm^2^ s^−1^	1.4399	±0.3212	±0.1618	±0.3930	0.88	0.12
			CV		22.3%	11.2%	27.3%		

**Table 5:** Reproducibility in volunteers for *D*. The table shows the mean and standard deviation values for the measured *D* together with the associated CV and ICC in grey matter (GM) and white matter (WM) in all scans, and in eight separate regions in 56 of the scans. All terminology is the same as for Table4

Volunteers				Mean	Inter-scanner (intra-volunteer)	Inter-volunteer (intra-scanner)	Intra-scanner (intra-volunteer)	ICC_inter_	ICC_intra_
DWI – IVIM – *D*	Overall	GM	×10^−3^ mm^2^ s^−1^	0.7495	±0.0207	±0.0186	±0.0159	0.63	0.37
			CV		2.8%	2.5%	2.1%		
		WM	×10^−3^ mm^2^ s^−1^	0.6506	±0.0249	±0.0115	±0.0108	0.84	0.16
			CV		3.8%	1.8%	1.7%		
	Cerebellum	Cortex (GM)	×10^−3^ mm^2^ s^−1^	0.7283	±0.0577	±0.0139	±0.0272	0.82	0.18
			CV		7.9%	1.9%	3.7%		
		WM	×10^−3^ mm^2^ s^−1^	0.6491	±0.0501	±0.0083	±0.0381	0.63	0.37
			CV		7.7%	1.3%	5.9%		
	Brain stem	All	×10^−3^ mm^2^ s^−1^	0.6819	±0.0665	NE	±0.0505	0.63	0.37
			CV		9.8%	NE	7.4%		
	Cerebrum	WM	×10^−3^ mm^2^ s^−1^	0.7035	±0.0294	±0.0098	±0.0157	0.78	0.22
			CV		4.2%	1.4%	2.2%		
		Basal ganglia	×10^−3^ mm^2^ s^−1^	0.7184	±0.0308	±0.0145	±0.0209	0.68	0.32
			CV		4.3%	2.0%	2.9%		
		Thalamus	×10^−3^ mm^2^ s^−1^	0.7367	±0.0040	±0.0178	±0.0213	0.78	0.22
			CV		5.4%	2.4%	2.9%		
		Choroid plexus	×10^−3^ mm^2^ s^−1^	1.8489	±0.2281	±0.1443	±0.2077	0.55	0.45
			CV		12.3%	7.8%	11.2%		
		Optic chiasm	×10^−3^ mm^2^ s^−1^	0.9730	±0.0755	±0.0962	±0.2815	0.07	0.93
			CV		7.8%	9.9%	28.9%		

**Table 6:** Reproducibility in volunteers for *f*. The table shows the mean and standard deviation values for the measured *f* together with the associated CV and ICC in grey matter (GM) and white matter (WM) in all scans, and in eight separate regions in 56 of the scans. All terminology is the same as for Table4

Volunteers				Mean	Inter-scanner (intra-volunteer)	Inter-volunteer (intra-scanner)	Intra-scanner (intra-volunteer)	ICC_inter_	ICC_intra_
DWI – IVIM – *f*	Overall	GM		0.1005	±0.0204	±0.0026	±0.0111	0.77	0.23
			CV		20.3%	2.6%	11.1%		
		WM		0.0799	±0.0234	±0.0020	±0.0047	0.96	0.04
			CV		29.2%	2.6%	5.8%		
	Cerebellum	Cortex (GM)		0.1441	±0.0244	±0.0080	±0.0121	0.80	0.20
			CV		16.9%	5.6%	8.4%		
		WM		0.0966	±0.0136	±0.0046	±0.0112	0.60	0.40
			CV		14.1%	4.8%	11.6%		
	Brain stem	All		0.1771	±0.0249	±0.0159	±0.0218	0.57	0.43
			CV		14.1%	9.0%	12.3%		
	Cerebrum	WM		0.0832	±0.0175	±0.0035	±0.0060	0.90	0.10
			CV		21.1%	4.2%	7.2%		
		Basal ganglia		0.0765	±0.0197	±0.0056	±0.0094	0.81	0.19
			CV		25.7%	7.3%	12.3%		
		Thalamus		0.1079	±0.0316	±0.0041	±0.0115	0.88	0.12
			CV		29.3%	3.8%	10.7%		
		Choroid plexus		0.2949	±0.0349	±0.0370	±0.0546	0.29	0.71
			CV		11.8%	12.6%	18.5%		
		Optic chiasm		0.4163	±0.1124	±0.0660	±0.1316	0.42	0.58
			CV		27.0%	15.8%	31.6%		

**Table 7:** Reproducibility in volunteers for MD. The table shows the mean and standard deviation values for the measured MD together with the associated CV and ICC in grey matter (GM) and white matter (WM) in all scans, and in eight separate regions in forty-seven of the scans. All terminology is the same as for Table4

Volunteers				Mean	Inter-scanner (intra-volunteer)	Inter-volunteer (intra-scanner)	Intra-scanner (intra-volunteer)	ICC_inter_	ICC_intra_
DTI – MD	Overall	GM	×10^−3^ mm^2^ s^−1^	0.8490	±0.0212	±0.0080	±0.0202	0.52	0.48
			CV		2.5%	0.9%	2.4%		
		WM	×10^−3^ mm^2^ s^−1^	0.6971	±0.0111	±0.0180	±0.0094	0.59	0.41
			CV		1.6%	2.6%	1.3%		
	Cerebellum	Cortex (GM)	×10^−3^ mm^2^ s^−1^	0.8506	±0.0824	±0.0212	±0.0446	0.77	0.23
			CV		9.7%	2.5%	5.2%		
		WM	×10^−3^ mm^2^ s^−1^	0.7209	±0.0523	±0.0100	±0.0393	0.64	0.36
			CV		7.3%	1.4%	5.5%		
	Brain stem	All	×10^−3^ mm^2^ s^−1^	0.9316	±0.0773	±0.0293	±0.0791	0.49	0.51
			CV		8.3%	3.1%	8.5%		
	Cerebrum	WM	×10^−3^ mm^2^ s^−1^	0.7851	±0.0328	±0.0142	±0.0236	0.66	0.34
			CV		4.2%	1.8%	3.0%		
		Basal ganglia	×10^−3^ mm^2^ s^−1^	0.8012	±0.0199	±0.0150	±0.0292	0.32	0.68
			CV		2.5%	1.9%	3.6%		
		Thalamus	×10^−3^ mm^2^ s^−1^	0.8846	±0.0618	±0.0394	±0.0871	0.33	0.67
			CV		7.0%	4.5%	9.9%		
		Choroid plexus	×10^−3^ mm^2^ s^−1^	2.2421	±0.3140	±0.1241	±0.2634	0.59	0.41
			CV		14.0%	5.5%	11.7%		
		Optic chiasm	×10^−3^ mm^2^ s^−1^	1.5959	±0.2154	±0.2592	±0.3011	0.34	0.66
			CV		13.5%	16.2%	18.9%		

**Table 8:** Reproducibility in volunteers for FA. The table shows the mean and standard deviation values for the measured FA together with the associated CV and ICC in grey matter (GM) and white matter (WM) in all scans, and in eight separate regions in forty-seven of the scans. All terminology is the same as for Table4

Volunteers				Mean	Inter-scanner (intra-volunteer)	Inter-volunteer (intra-scanner)	Intra-scanner (intra-volunteer)	ICC_inter_	ICC_intra_
DTI – FA	Overall	GM	×10^−3^ mm^2^ s^−1^	0.1726	±0.0047	±0.0097	±0.0128	0.12	0.88
			CV		2.7%	5.6%	7.4%		
		WM	×10^−3^ mm^2^ s^−1^	0.4187	±.0083	±0.0157	±0.0088	0.47	0.53
			CV		2.0%	3.8%	2.1%		
	Cerebellum	Cortex (GM)	×10^−3^ mm^2^ s^−1^	0.1991	±0.0142	±0.0077	±0.0201	0.33	0.67
			CV		7.1%	3.9%	10.1%		
		WM	×10^−3^ mm^2^ s^−1^	0.3872	±0.0253	±0.0204	±0.0269	0.47	0.33
			CV		6.5%	5.3%	6.9%		
	Brain stem	All	×10^−3^ mm^2^ s^−1^	0.4139	±0.0224	±0.0157	±0.0243	0.46	0.54
			CV		5.4%	3.8%	5.9%		
	Cerebrum	WM	×10^−3^ mm^2^ s^−1^	0.3275	±0.0529	±0.0102	±0.0254	0.81	0.19
			CV		16.1%	3.1%	7.8%		
		Basal ganglia	×10^−3^ mm^2^ s^−1^	0.2351	±0.0373	±0.0159	±0.0247	0.69	0.31
			CV		15.8%	6.8%	10.5%		
		Thalamus	×10^−3^ mm^2^ s^−1^	0.2879	±0.0090	±0.0151	±0.0220	0.14	0.86
			CV		3.1%	5.2%	7.6%		
		Choroid plexus	×10^−3^ mm^2^ s^−1^	0.1592	±0.0321	NE	±0.042	0.37	0.63
			CV		20.1%	NE	26.4%		
		Optic chiasm	×10^−3^ mm^2^ s^−1^	0.1781	±0.0377	NE	±0.0857	0.16	0.84
			CV		21.2%	NE	48.1%		

Considering overall grey matter and white matter, ADC, *D*, MD and FA showed an intra-scanner and inter-scanner CV ranging between 1% (ADC in white matter) and 7.4% (FA in grey matter), with a mean CV of 2.6%. The reproducibility of *f* was lower than for the other parameters, with an average intra-scanner CV of 8.4% and inter-scanner CV of 24.8%. Similarly to the results in the phantom, it was noted that the value of *f* in scanner G was much higher than in the other scanners, as also seen in the boxplots in Figures4 and 5, and excluding this scanner reduced the inter-scanner reproducibility to 6.9%.

The mean ADC, *D* and MD were 0.84, 0.75 and 0.85 × 10^−3^ mm^2^ s^−1^ in grey matter and 0.7, 0.65 and 0.7 × 10^−3^ mm^2^ s^−1^ in white matter, respectively. The mean value of *f* was 0.1 in grey matter and 0.08 in white matter. Excluding scanner G, the mean value of *f* was 0.093 in grey matter and 0.072 in white matter. The mean FA was 0.17 in grey matter and 0.42 in white matter.

The reproducibility of ADC, *D*, MD and FA was worse in the specific brain regions analysed as compared with overall grey and white matter, and the highest CV was found in the choroid plexus and the optic chiasm. The reproducibility of FA in specific white matter areas is shown in Table9. The mean intra- and inter-scanner CV was 4.2% and 4.4% respectively, with a mean FA ranging from 0.43 to 0.65 depending on the areas analysed.

**Table 9:** Reproducibility of FA. The table shows the mean and standard deviation for FA, together with the associated CV and ICC, as measured in each of the specified white matter areas. All terminology is the same as for Table4

Volunteers			Mean	Inter-scanner (intra-volunteer)	Inter-volunteer (intra-scanner)	Intra-scanner (intra-volunteer)	ICC_inter_	ICC_intra_
DTI – FA	Genu of corpus callosum		0.6257	±0.0195	±0.0226	±0.0154	0.62	0.38
		CV		3.1%	3.6%	2.5%		
	Body of corpus callosum		0.5838	±0.0199	±0.0260	±0.0143	0.66	0.34
		CV		3.4%	4.4%	2.5%		
	Splenium of corpus callosum		0.6523	±0.0168	±0.0493	±0.0165	0.51	0.49
		CV		2.6%	7.6%	2.5%		
	Cerebral peduncle – right		0.5966	±0.0314	±0.0493	±0.0235	0.64	0.36
		CV		5.3%	8.3%	3.9%		
	Cerebral peduncle – left		0.6059	±0.0378	±0.0407	±0.0255	0.69	0.31
		CV		6.2%	6.7%	4.2%		
	Sagittal stratum – right		0.5091	±0.0241	±0.0359	±0.0168	0.67	0.33
		CV		4.7%	7.0%	3.3%		
	Sagittal stratum – left		0.4975	±0.0158	±0.0379	±0.0150	0.53	0.47
		CV		3.2%	7.6%	3.0%		
	Cingulum (hippocampus) – right		0.4289	±0.0266	±0.0292	±0.0397	0.31	0.69
		CV		6.2%	6.8%	9.2%		
	Cingulum (hippocampus) – left		0.4514	±0.0200	±0.0439	±0.0399	0.20	0.80
		CV		4.4%	9.7%	8.8%		
	Superior longitudinal fasciculus – right		0.4462	±0.0224	±0.0319	±0.0132	0.74	0.26
		CV		5.0%	7.1%	3.0%		
	Superior longitudinal fasciculus – left		0.4400	±0.0185	±0.0158	±0.0136	0.65	0.35
		CV		4.2%	3.6%	3.1%		

### Field strength comparison

**Figure 7:**
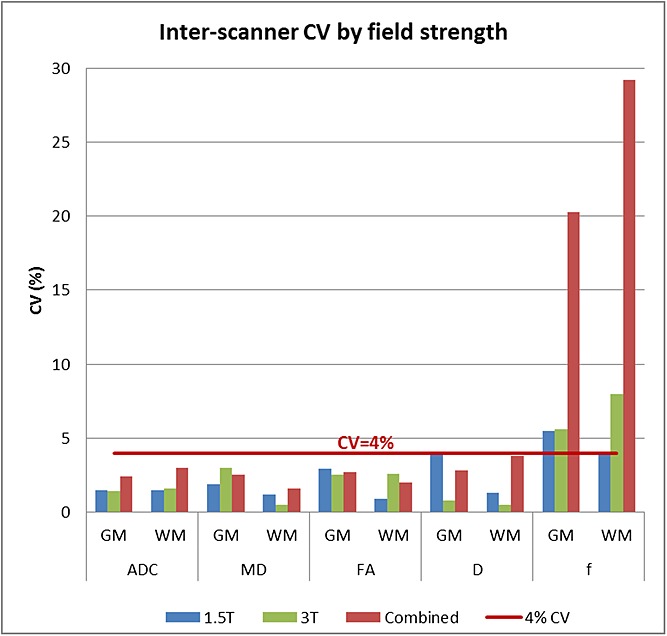
Inter-scanner CV by field strength. A comparison of the inter-scanner CV in overall grey matter (GM) and overall white matter (WM) for the model constructed using only 1.5T scanners, only 3T scanners and both field strengths is shown. The CV was less than 4% for all parameters except for the perfusion fraction.

When fitting the mixed-effect model to the 1.5T and 3T scanners separately, we did not observe any consistent pattern for differences between the two field strengths. Results for the CV from the two scanner field strengths are shown separately and combined in Figure7 and Table10.

**Table 10:** Comparison of CV in 1.5, 3T and all scanners. The table shows the inter-scanner and the intra-scanner CV when 1.5 and 3T scanners are analysed separately for GM and WM regions, together with the reported CV when combining all scanners together. A larger difference in inter-scanner CV can be seen for *f* when combining data from all scanners together. No large differences in CV can be seen otherwise

			Inter-scanner			Intra-scanner		
Field strength			1.5T	3T	All	1.5	3	All
ADC	GM	CV (%)	1.5	1.4	2.4	2.6	3.5	2.9
	WM		1.5	1.6	3.0	1.3	0.7	1.0
*D*	GM		3.9	0.8	2.8	1.4	2.5	2.1
	WM		1.3	0.5	3.8	0.8	1.0	1.7
*f*	GM		5.5	5.6	20.3	7.5	13.9	11.1
	WM		4.1	8.0	29.2	2.3	5.4	5.8
MD	GM		1.9	3.0	2.5	1.9	2.5	2.4
	WM		1.2	0.5	1.6	0.9	1.4	1.3
FA	GM		2.9	2.5	2.7	7.2	1.6	7.4
	WM		0.9	2.6	2.0	1.6	2.0	2.1

## Discussion

Multi-centre studies are becoming increasingly important with the discovery of new genetic biomarkers that characterize specific and rare disease types. In such cases, patients with specific rare diseases could be grouped and studied together across centres, leading to larger sample sizes and more powerful data analyses. Furthermore, meaningful use of imaging biomarkers for treatment stratification and prognostication is dependent on robust interpretation of data from multiple centres. The reproducibility of both DWI and DTI parameters was studied to compare diffusion imaging across centres. Whilst protocols were agreed prior to the study initiation, local variation is seen in the implemented protocols, to allow inclusion of historically acquired data, and it is important to determine whether data can be combined from such datasets.

### Phantom

The diffusion coefficient of water, at 0 °C, in the ice phantom is expected to be 1.099 × 10^−3^ mm^2^ s^−1^ (15). The phantom is not perfused and hence the perfusion fraction, *f*, is expected to be close to zero, and the result of noise or model fitting errors. The IVIM model is also therefore expected to have a slow diffusion coefficient, *D*, similar to the ADC. MD is expected to have a value comparable to the ADC (ADC was measured using DWI as an average of three directions; MD was measured from the diffusion tensor with data acquired over 15–60 directions). FA is expected to be close to zero due to free diffusion in the water-filled tubes, and therefore its calculated value is dominated by the effects of noise and a high CV is consequently expected.

ADC, *D* and MD values were similar and close to the expected value of 1.099 × 10^−3^ mm^2^ s^−1^ in all scanners except for scanner F, where the ice phantom was not given sufficient time to reach thermal equilibrium. A phantom that is stable over time would be a valuable aid to multi-centre studies by providing a tool for assessing differences expected between scanners and over time, and hence giving a measure for the inherent variation expected in the multi-centre data acquired. The three parameters were reproducible with a CV of less than 1.5% when scanner F was excluded. The low reproducibility of FA was expected. Magnitude images have a rectified noise floor (28), which leads to non-zero calculation of FA even in an isotropic medium, and hence is system dependent. Similarly, the low reproducibility of *f* was expected, as it is a representation of both noise and over-fitting a bi-exponential to data that is in fact mono-exponential. The mean value of *f* was 0.02 and that of FA was 0.04. While the use of an anisotropic phantom would have been more relevant for an assessment of anisotropy, such a phantom was not available for this study. However, progress has been made in developing anisotropic phantoms (29), which could be used in future multi-centre analyses of FA.

### Volunteers

#### ADC, D and MD

In volunteers, ADC and MD are again expected to yield similar results, with *D* having a similar but lower value due to the IVIM calculation incorporating a perfusion component. Furthermore, the diffusion coefficient of grey matter is expected to be higher than that of white matter (30). Our results were consistent with these findings; with ADC and MD giving similar results for a value of 0.84 × 10^−3^ mm^2^ s^−1^ and 0.85 × 10^−3^ mm^2^ s^−1^ in grey matter respectively and 0.70 × 10^−3^ mm^2^ s^−1^ for white matter in both cases. *D* showed lower mean values of 0.75 × 10^−3^ mm^2^ s^−1^ and 0.65 × 10^−3^ mm^2^ s^−1^ in grey and white matter respectively. ADC, *D* and MD all had a good reproducibility in both white matter and grey matter, with a mean CV of 2.3%.

#### Perfusion fraction, f

The higher value of *f* in grey compared with white matter reflects the increased vascularity and perfusion of grey matter. Our results showed the mean values to be 0.10 and 0.08 in grey and white matter respectively, which are concordant with values found in previous literature, where *f* in grey and white matter was found to be 0.11 and 0.076 respectively (31). However, due to factors such as partial volume fractions and differences in relaxation times between grey matter, white matter and blood (5,32), these values are not a direct measure of cerebral blood volume fractions (approximately 5.2% and 2.7% in grey and white matter respectively (33)). The significantly higher value of *f* in both grey and white matter compared with the ice–water phantom demonstrates that perfusion effects have a significant effect on the bi-exponential component of raw DWI data acquired *in vivo*.

The reproducibility of *f* was found to be low overall, but the CV improved to 5.3% in grey matter and 8.5% in white matter by excluding the results from scanner G, which had consistently higher *f* for both the phantom and volunteers. It is likely that the higher estimation of *f* in scanner G could be driven by the higher acquisition resolution in this scanner, as compared with the other scanners, implying a lower signal to noise ratio and, as shown in previous work, an increase in *f* (31).

#### Fractional anisotropy, FA

FA is highest in white matter, where the presence of structured fibres contributes to the anisotropy of the diffusion of water molecules. Measured FA was higher in white matter, as expected, with a mean value of 0.42 as compared with that found in grey matter of 0.17. FA had a good reproducibility, with an inter-scanner CV of 2% when measured over all the white matter regions and a higher mean inter-scanner CV of 4.3% when measured in specific white matter tracts.

#### Overall reproducibility

Overall, the inter-scanner reproducibility of ADC, *D*, MD and FA in total brain grey and white matter was less than 4% in all scanners. Variations from scanning the same person on a different scanner (the inter-scanner reproducibility) may be expected to be higher than those from scanning the same person on the same scanner (the intra-scanner reproducibility), as the variability between scanner systems is likely to have a larger effect on the measured reproducibility. From our results, the intra-scanner reproducibility was usually better than the inter-scanner reproducibility. However, intra-scanner variations include any errors that are not related to scanner or person differences such as image noise and data processing errors, and, e.g., in the case of FA, the intra-scanner variation was worse than the inter-scanner variation in grey matter. This may be a result of the lower FA values seen in grey matter (closer to zero) having an increased effect from noise and worse intra-scanner reproducibility.

The differences between scanner field strengths were assessed by analysing the data separately for the 1.5T and the 3T scanners. This process was used as there was insufficient data to include field strength as one of the effects in the mixed-effect model. Analysing the two scanner field strengths separately yielded similar results between 1.5 and 3T scanners, with the overall inter-scanner CV being comparable for all parameters except for *f*. This implies that the error associated with using data from the different scanners analysed, both 1.5T and 3T, would have a similar impact as using data from the same scanner, and hence supports the use of ADC, *D*, MD and FA data from across the different scanners.

Similar results to ours were observed by Veenith *et al.* (6), where the intra-scanner CV for MD and FA was reported to be less than 6%, by Magnotta *et al.* (11), where the inter-scanner CV was reported to be less than 3.2% in both MD and FA, by Pfefferbaum *et al.* (7), where the inter-scanner variation was reported to be less than 3.8% for both MD and FA in supratentorial white matter, and by Vollmar *et al.* (9), where the intra-scanner CV was reported to be less than 3% and the inter-scanner CV to be less than 4.1% for FA. The inter-scanner reproducibility of ADC in the work of Sasaki *et al.* (8) was calculated from the minimum and maximum mean differences and ADC was shown to vary between 3.8 and 8.8% across both grey and white matter. The study showed a poorer reproducibility than calculated in our study, and could be attributed to the method used – where in the work of Sasaki *et al.* (8) grey matter was considered by drawing an ROI around the thalamus, and white matter was considered by drawing an ROI around the bilateral frontal white matter. When analysing the thalamus separately, our study showed an inter-scanner CV of 10.2%, a result more comparable to that of (8). In addition to these analyses, Cercignani *et al.* (4) reported an inter-scanner CV between two scanners of 5.4% for MD and 7.7% for FA using a histogram-based approach, which did not separate grey matter from white matter regions and hence cannot be directly compared with the results of the study presented here.

#### Analysing specific brain regions

The reproducibility of diffusion imaging parameters was lower in specific brain regions, compared with that in overall grey and white matter areas. This may be due to the lower number of voxels being analysed and errors associated with the introduction of a registration step. The CV was particularly low in the choroid plexus and the optic chiasm, which may reflect the small areas studied. These areas are also surrounded by CSF, making them particularly susceptible to partial volume effect. They were included as they are known sites for paediatric brain tumours, although tumours are much larger than the structures from which they arise and may well provide more reproducible data. Partial volume is highlighted by the high value of *f* in these regions, as a mixed population of diffusing species increases the biexponential behaviour of the DWI signal. Excluding these regions, the maximum inter-scanner CV was 14.2%, 9.8%, 9.7% and 16.1% for ADC, *D*, MD and FA, with a mean CV of 11.3%, 6.5%, 6.5% and 9% respectively. In addition, the lower reproducibility of FA when studied in specific regions of the brain may be due to the inherent errors that exist in using a standard brain mask and registration. Nonetheless, the CV in each of the areas studied was less than 10%, with a mean CV of 4.3% for both intra- and inter-scanner differences. This is in agreement with previous studies: in the work of Teipel *et al.* (10) a CV of 7.4% was reported for FA in white matter and 8.4% for FA in grey matter when scanning one volunteer across 14 scanners by segmenting *T*_1_-weighted images, and in the work of Pfefferbaum *et al.* (7) a CV of less than 8.7% was reported when analysing a specific brain region (in that case the corpus callosum). Furthermore, although the CV was higher when analysing specific brain areas, ICC_inter_ in these regions was similar to the values in overall grey and white matter in most cases. This may be an indication that the increase in error is likely to be due to the method of analysis itself, rather than the variations between scanners. Thus, the lower reproducibility of data from specific brain regions highlights the care needed when involving more image processing steps such as registration, and when segmenting smaller regions.

#### Advantages

The current study has comparable reproducibility to previous studies *but it has used clinical sequences not previously matched across individual scanners, providing evidence that large multi-centre studies could be undertaken without extensive harmonization of protocols*. This supports, to a degree, the inclusion of previously acquired retrospective data in multi-centre studies, although reproducibility analysis across a given group of scanners with differing protocols is advised. The implications of this study suggest that the difficulty of individual centres being required to alter their standard clinical sequence parameters may be overcome. Otherwise, specialized protocols different from those in routine clinical use would preclude the use of historically acquired data. The ability to use clinical sequence parameters across sites is a desirable factor allowing potentially more centres to contribute data for multi-centre analysis. Furthermore, the study encompassed a range of scanners: four 1.5T and four 3T scanners, supporting the use of longitudinal diffusion imaging data from scanners of different field strengths.

The CV can be used as a way of determining the minimum diffusion parameter changes required in order to be able to correlate changes in imaging with other clinically relevant measures, such as treatment response or prognosis of specific tumour subtypes. We recommend that the minimum change in a parameter value be of at least two standard deviations, and hence 2 × CV, in order to be considered as a change that is not related to inter-scanner differences. Thus a change of more than 8% in the value of ADC, *D*, MD or FA in overall white matter, between e.g. two different time-points, is likely to be due to differences other than scanner variability. While a change in such neurological conditions as stroke and tumours may be large enough to be analysed using different clinical sequences, other neurological disorders may show more subtle variations that may require more stringent conditions in terms of scanner and acquisition protocol variability. Thus the importance of having matching imaging parameters is also pathology dependent, and the effect size under analysis must be taken into consideration in determining whether the reproducibility, both inter- and intra-scanner, is sufficiently high for a given analysis.

### Limitations

The main limitation of the study lies in the image registration method and in the segmentation method of white matter, grey matter and each of the different brain regions analysed, which may be prone to partial volume effects. In order to limit the partial volume effect, only those voxels with a probability higher than 0.95 were accepted for use as grey and white matter and segmented areas were eroded by one voxel. Some harmonization of the protocols at the centres had been undertaken, and so it should not be assumed that the parameters would have the same CVs if data were taken from a set of clinical centres without any guidance in the protocols to be used. For example, the protocol advised that *b*-values of 0 and 1000 should be included in the protocols and, previous studies have shown the maximum *b*-value to have an impact on the measured ADC (34,35). Hence the impact of using a different upper range of *b*-values was not studied here. In addition, isotropic or near isotropic voxels were recommended for the DTI sequence and protocols that have a much higher in plane resolution than slice thickness, which are the default on some scanners, may result in a higher CV for FA. Finally, due to practical and ethical considerations, the current study was carried out in healthy volunteers; however, a study in patients would ideally be conducted to measure the effect of the specific pathology on the imaging reproducibility.

## Conclusion

Diffusion MRI measures, in particular ADC, *D*, MD and FA, have a good reproducibility across both 1.5T and 3T scanners. Quantitative research studies can benefit from incorporating multi-centre data using clinical sequences and protocols without any significant loss of reproducibility compared with that achieved from a single scanner at a single site.

## Abbreviations

ADC: apparent diffusion coefficient
CV: coefficient of variation
DTI: diffusion tensor imaging
DWI: diffusion-weighted imaging
FA: fractional anisotropy
ICC: inter-class correlation coefficient
ICC_intra_: intra-class correlation coefficient
IVIM: intra-voxel incoherent motion
MD: mean diffusivity
ROI: region of interest.
